# IMP-ICDX: an injury mortality prediction based on ICD-10-CM codes

**DOI:** 10.1186/s13017-019-0265-y

**Published:** 2019-10-11

**Authors:** Muding Wang, Wusi Qiu, Yunji Zeng, Wenhui Fan, Xiao Lian, Yi Shen

**Affiliations:** 1 Department of Emergency Medicine, Affiliated Hospital of Hangzhou Normal University, 126 Wenzhou Road, Gongshu District, Hangzhou, 310015 Zhejiang People’s Republic of China; 2 Department of Neurosurgery, Affiliated Hospital of Hangzhou Normal University, Hangzhou, 310015 Zhejiang People’s Republic of China; 3 Department of Orthopedic, Affiliated Hospital of Hangzhou Normal University, Hangzhou, 310015 Zhejiang People’s Republic of China; 4 Department of Epidemiology and Health Statistics, School of Public Health, Zhejiang University, Hangzhou, 310058 Zhejiang People’s Republic of China

**Keywords:** International Classification of Diseases Tenth Edition (ICD-10-CM), Injury mortality prediction for ICD-10-CM (IMP-ICDX), Injury severity score (ISS), Mortality prediction

## Abstract

**Background:**

The International Classification of Diseases, Ninth Edition, Clinical Modification (ICD-9-CM) Injury Severity Score (ICISS) is a risk adjustment model when injuries are recorded using ICD-9-CM coding. The trauma mortality prediction model (TMPM-ICD9) provides better calibration and discrimination compared with ICISS and injury severity score (ISS). Though TMPM-ICD9 is statistically rigorous, it is not precise enough mathematically and has the tendency to overestimate injury severity. The purpose of this study is to develop a new ICD-10-CM injury model which estimates injury severities for every injury in the ICD-10-CM lexicon by a combination of rigorous statistical probit models and mathematical properties and improves the prediction accuracy.

**Methods:**

We developed an injury mortality prediction (IMP-ICDX) using data of 794,098 patients admitted to 738 hospitals in the National Trauma Data Bank from 2015 to 2016. Empiric measures of severity for each of the trauma ICD-10-CM codes were estimated using a weighted median death probability (WMDP) measurement and then used as the basis for IMP-ICDX. ISS (version 2005) and the single worst injury (SWI) model were re-estimated. The performance of each of these models was compared by using the area under the receiver operating characteristic (AUC), the Hosmer-Lemeshow (HL) statistic, and the Akaike information criterion statistic.

**Results:**

IMP-ICDX exhibits significantly better discrimination (AUC_IMP-ICDX_, 0.893, and 95% confidence interval (CI), 0.887 to 0.898; AUC_ISS_, 0.853, and 95% CI, 0.846 to 0.860; and AUC_SWI_, 0.886, and 95% CI, 0.881 to 0.892) and calibration (HL_IMP-ICDX_, 68, and 95% CI, 36 to 98; HL_ISS_, 252, and 95% CI, 191 to 310; and HL_SWI_, 92, and 95% CI, 53 to 128) compared with ISS and SWI. All models were improved after the extension of age, gender, and injury mechanism, but the augmented IMP-ICDX still dominated ISS and SWI by every performance.

**Conclusions:**

The IMP-ICDX has a better discrimination and calibration compared to ISS. Therefore, we believe that IMP-ICDX could be a new viable trauma research assessment method.

## Introduction

Trauma score methods can be divided into two categories of systems. First, the injury severity score (ISS), the new injury severity score (NISS), the tangent injury severity score (TISS), the trauma mortality prediction model (TMPM), and injury mortality prediction (IMP) [1–5] score methods based on the Abbreviated Injury Scale (AIS) [6] lexicon. Their ability of predicting trauma death is also improved [2–5, 7]. However, the AIS codes must be evaluated by trauma surgeon experts. In these circumstances, a great deal of manpower and material resources is consumed. It is difficult for developed countries, let alone developing ones. These situations hinder the trauma score in-depth research and popularization. Second, the International Classification of Diseases Ninth Edition (ICD-9-CM) Injury Severity Score (ICISS) and the trauma mortality prediction model (TMPM)-ICD9 score methods based on ICD-9-CM lexicon [8, 9]. ICD-9-CM codes are the common disease diagnosis codes around the world. Currently, most countries and regions apply the updated ICD-10-CM. The number of diagnostic categories available is approximately over 9000, which is more than the number of AIS code categories. Although ICD-10-CM codes are not similar to AIS which implies injury severities, each diagnosis has implied the information of anatomy trauma, a variety of disease severity, and the possibility of mortality. ICD-10-CM codes also include the possibility of death, such as traumatic hemorrhage of right cerebrum with loss of consciousness of 30 min or less, initial encounter; displaced fracture of base of neck of right femur, initial encounter for closed fracture; and major laceration of liver, initial encounter.

The ICISS is the product of empirically derived survival risk ratios (SRRs) for trauma ICD-9-CM codes [8]. SRR is a survival rate of all trauma patients in a specific trauma ICD-9-CM code. It contains survival rates of patients who sustained both a single injury and multiple injuries. Although ICISS is better than the ISS and NISS in the prediction ability of death [8, 10, 11], the SRR underestimates the survival rate of patients with a single injury and overestimates survival rate of patients with multiple injuries. Therefore, ICISS is inaccurate for the prediction of mortality (survival).

TMPM-ICD9 [9] derived an empirical severity value for each ICD-9-CM code that is called the model-averaged regression coefficient (MARC) which is similar to TMPM [4]. Then, calculating the TMPM-ICD9 value according to MARC values by using a special formula. The TMPM-ICD9 is better than the ICISS as a predictor of mortality [7, 9]. Researchers concluded that the TMPM-ICD9 outperforms the ISS and NISS in mortality prediction [7, 12]. TMPM-ICD9 is statistically rigorous, but it is not accurate enough in mathematics. There is a tendency to overestimate the severity of the injury [12].

We propose a new ICD-10-CM injury model which replaces the sole regression-based approach. Then we compare the performance of injury mortality prediction (IMP-ICDX), a new mortality prediction model based on these empiric injury severities, with ISS and single worst injury (SWI) models. Our objective was that the IMP-ICDX would provide a more accurate prediction of mortality than other existing scoring systems.

## Methods

### Data source

**Fig. 1:**
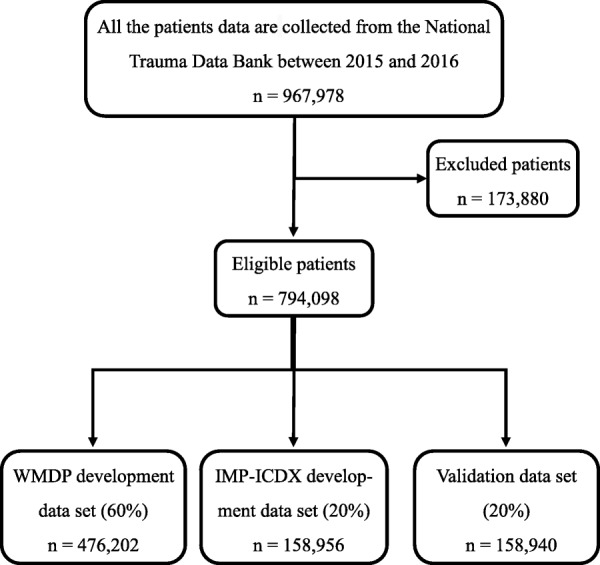
Flowchart for data analyzed

The patients came from the National Trauma Data Bank (NTDB) hospitalized between 2015 and 2016. Available information included patient demographics, ICD-10-CM diagnostic and injury codes (national clinical revision in American), mechanism of injury (according to ICD-10-CM E-codes), ISS (version 2005), in-hospital mortality, Glasgow Coma Score (GCS), and encrypted hospital identifiers. This dataset consisted of 967,978 patients with 1 or more ICD-10-CM injury codes and AIS codes. Patients with non-traumatic diagnoses (e.g., drowning, poisoning, and suffocation) or burns (47,184), missing or invalid data (data missing on length of hospital stay, age, gender, or outcome) (26,177), missing cause of trauma (8938), or age younger than 1 year (3900) and older than 89 years (60,917) were excluded from our analysis. The reason is that patients over the age of 89 were a separate age category in the NDTB and were assigned the value of − 99 for their age. Patients who transferred to another facility (37,014) or were dead on arrival to the hospital (10,388) were also excluded. Some patients were excluded from the analysis because they have more than 1 exclusion criteria. ICD-10-CM E-codes were mapped to 1 of the 6 injury mechanisms by an experienced trauma surgeon: fall, motor vehicle crash, violence, gunshot wound, stab wound, and blunt injury. The final dataset included 794,098 patients admitted to 738 trauma centers. The details for recruitment are shown in Fig. 1.

### Overview of IMP-ICDX development

In this research, 60% of the total dataset is used to evaluate trauma mortality rate (TMR) of different ICD-10-CM codes. The TMR values are calculated in Additional file 1. Based on TMR, number of body region (NBR), and body region (BR), we created three separate probit regression models by adding six additional variables: age, gender, GCS, ventilator, mechanism of injury, and hospital fixed effects to each of them. In the meantime, we applied optimal ratio of death probability for NBR and BR to modify the traumatic death probability (TDP) for TMR, to achieve an optimal value. The median of the three greatest (worst) TDP values was adopted as a weighted median death probability (WMDP) for each specific ICD-10-CM code (see Additional file 2).

Twenty percent of the dataset (IMP-ICDX development dataset) is used to evaluate IMP-ICDX. We apply logistic regression model to calculate coefficient of IMP-ICDX (Table 4) and deduce specific formula for the IMP-ICDX (see Additional file 3). Twenty percent of the dataset (internal validation dataset) is not used for the development of WMDP and IMP-ICDX to estimate the statistical performance of IMP-ICDX.

### Customization of trauma models

This internal validation dataset enables us to test the performance of the ISS, SWI, and IMP-ICDX. ISS was computed according to Baker et al. [1]. A single worst injury (SWI) model was defined as the WMDP value for the worst injury (i.e., the greatest WMDP value). IMP-ICDX comprises the five most severe WMDP values according to injury severity; the product of the WMDP values for the two worst injuries is used as a variable and determines whether or not the two worst injuries are in the same BR and NBR (as ln (NBR) and NBR^0.382^, suggested by fractional polynomial analysis [13]) of each individual injury patient. The probability of death was calculated with the specific IMP-ICDX formula. At the same time, we then re-estimate all three models after adding age, gender, and injury mechanism to simple injury models, which only include the information on anatomic injury. Robust variance estimators [14] were applied because of the possible correlate outcomes of patients treated at the same trauma center.

### Statistical analysis

This article assessed the statistical performance of all models using the area under the receiver operating characteristic (AUC) curve for discrimination, the Hosmer-Lemeshow (HL) statistic for calibration, and the Akaike information criterion (AIC) for proximity to the true model. Non-parametric bootstrapping resampling algorithm with 1000 replications provided 95% confidence intervals (CIs) for the AUC and HL statistic. A *P* < 0.05 was considered statistically significant. All statistical analyses were performed using STATA/MP version 14.0 for Windows. This paper was exempt from review by the Institutional Review Board of Hangzhou Normal University, People’s Republic of China.

## Results

In this text, the total of the WMDP values is 8534 different ICD-10-CM coded injuries (see Additional file 4). These WMDP values range from 0.009 for a minor injury (ICD-10-CM, S42.412A: “Displaced simple supracondylar fracture without intercondylar fracture of left humerus, initial encounter for closed fracture”) to a value of 1.927 for a severe injury (ICD-10-CM, S06.5X7A: “Traumatic subdural hemorrhage with loss of consciousness of any duration with death due to brain injury, initial encounter”). Although trauma ICD-10-CM codes are not set by experts and cannot show information of traumatic severity, which are different from AIS codes, this research calculates the WMDP values of different ICD-10-CM codes and uses them to react to the degree of severity of trauma. We believe that these WMDP values are appropriate and in accordance with the actual situation of clinical, not our subjective assume.

Patient demographics are summarized in Table 1. The median age of our cohort was 49 years. Males accounted for 61.3%, and 66.4% was non-Hispanic White. The majority of patients in this text were fall (44.4%) and motor vehicle collisions (35.8%). The overall mortality rate for the patients was 2.41%.

**Table 1:** Patient demographics

Patient characteristics	No. of patients (%)
Demographic variables	
Age, median (IQR)	49 (26–69)
Gender (male)	486,930 (61.3)
Mechanism of injury	
Fall	352,479 (44.4)
Motor vehicle accident	284,329 (35.8)
Violence^*^	59,906 (7.6)
Gunshot	35,883 (4.5)
Stab	35,234 (4.4)
Blunt	26,267 (3.3)
Dead	19,145 (2.41)

*IQR* interquartile range

^*^Violence means to strike or against

**Fig. 2:**
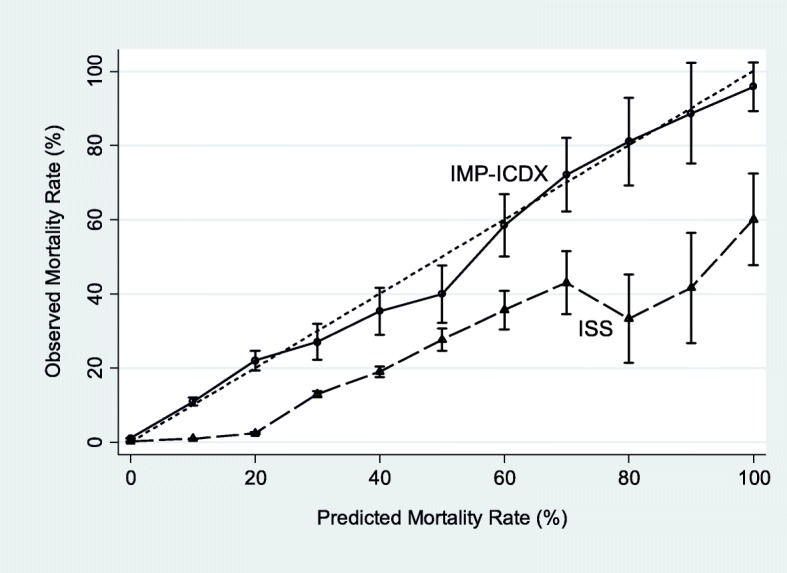
Calibration curves for IMP-ICDX and ISS. The dotted reference lines represent perfect calibration (95% binomial confidence intervals for IMP-ICDX and ISS models are based on the same validation dataset of 158,940 patients)

The statistical performance of all models is shown in Tables 2 and 3. The IMP-ICDX displays significantly better discrimination, calibration, or AIC statistic compared with both the ISS and SWI models. Figure 2 graphically displays the superior calibration of IMP-ICDX. The ISS values were distributed to the right of the dotted reference line. The IMP-ICDX values were uniformly distributed much closer to the dotted reference line. The IMP-ICDX coefficients are shown in Table 4.

**Table 2:** Model performance: anatomic injury models

Model description	AUC (95% CI)	H-L stat (95% CI)	AIC
ISS	0.853 (0.846–0.860)	252 (191–310)	27,655
Single worst injury	0.886 (0.881–0.892)	92 (53–128)	23,289
IMP-ICDX	0.893 (0.887–0.898)	68 (36–98)	23,024

The IMP-ICDX demonstrated the best discrimination, calibration, and AIC compared to the single worst injury and ISS models

**Table 3:** Model performance: anatomic injury models augmented with age, gender, and injury mechanism

Model description	AUC (95% CI)	H-L stat (95% CI)	AIC
ISS	0.903 (0.899–0.908)	152 (106–202)	25,916
Single worst injury	0.915 (0.910–0.919)	37.4 (18.3–52)	21,970
IMP-ICDX	0.919 (0.914–0.923)	26.5 (11.2–41)	21,660

Although every model will be changed by the addition of more predictors, IMP-ICDX still shows superior model compared to the SWI and ISS models

**Table 4:** IMP-ICDX regression coefficients

Predictor		Coefficient	Robust std. error	*Z*	*P* > |*Z*|	95% CI
WMDP_1_	C_1_	4.9695	0.1198	41.49	0.000	4.7348–5.2043
WMDP_2_	C_2_	2.2315	0.2467	9.04	0.000	1.7479–2.7151
WMDP_3_	C_3_	0.4111	0.1215	3.38	0.001	0.1730–0.6492
WMDP_4_	C_4_	0.3107	0.1418	2.19	0.028	0.0328–0.5885
WMDP_5_	C_5_	0.3905	0.1318	2.96	0.003	0.1322–0.6488
WMDP_1_ × WMDP_2_	C_6_	− 0.7551	0.1428	− 5.29	0.000	− 1.0349 to − 0.4753
Same region	C_7_	− 0.2120	0.0488	− 4.34	0.000	− 0.3076 to − 0.1163
ln (NBR)	C_8_	− 2.4871	0.1942	− 12.80	0.000	− 2.8678 to − 2.1064
NBR^0.382^	C_9_	2.6147	0.2331	11.22	0.000	2.1578–3.0715
Constant	C_0_	− 10.7138	0.2630	− 40.73	0.000	− 11.2293 to − 10.1983

Coefficients for IMP-ICDX model were recalculated based on all 158,956 patients not used to calculate WMDP and IMP-ICDX values. WMDP_1_ indicates the worst injury (greatest WMDP value), WMDP_2_ the second worst injury, and so on. WMDP_1_ × WMDP_2_ is the product of the WMDP values for the 2 worst injuries. Same region is equal to 1 if the two worst injuries are in the same body region, 0 otherwise. NBR is the number of body regions for each injured patient. “ln” indicates natural logarithm

## Discussion

The probability of death from patient trauma depends on many factors. The most important condition is the patient’s trauma severity. With the progress of medical science and the improvement of the treatment level, the trauma mortality has decreased obviously. Most of the existing trauma scores are difficult to distinguish real severity of all trauma patients, and even if there are trauma patients with similar severity, the results of treatment in different hospitals are also significantly differences [15]. This research also has similar results. For any individual patients, the likelihood of death is always accompanied by the whole course of treatment.

At present, there are many trauma score methods. For instance, ISS, NISS, and TISS are rapid evaluation methods while TMPM and IMP are retrospective evaluation methods, and they are all based on AIS codes. These methods have been widely used in clinical practice. They require that all patients have their injuries described in the AIS lexicon. Otherwise, they cannot be used to calculate, which limits their application. The ICISS and IMPM-ICD9, which are based on ICD-9-CM code, have broken away from the AIS code and opened up a new way of scoring method. TMPM-ICD9 is better than ICISS in predicting death results [7, 9]. The data used in this study was derived from ICD-10-CM instead of ICD-9-CM. The above scoring methods are not suitable. Though ICD-10-CM encoding can be converted to ICD-9-CD code and AIS code can be generated, the result after conversion is bound to be biased. It is not in line with the original intention of this research. Therefore, it is sensible to compare IMP-ICDX with ISS in our study.

This text combines the large dataset of NTDB and the feasible scoring method to evaluate the results of the trauma. The NTDB has the world’s largest and the most credible trauma dataset and contains trauma data of different trauma centers in different regions of the USA. It includes information that offers us with research.

In this TMR development dataset, when the actual mortality rate of specific ICD-10-CM code is 0, the TMR value is based on the death trend of the National Vital Statistics Reports in the United States in 2015 [16]. It is set as the median of the possible mortality rate (PMR_M) ( see Additional file 1) because the data is not normally distributed. There are 105 (only contains 370 patients) single or multiple injuries with 100% mortality, but these single or multiple injuries each has 80 or fewer cases, and there is only 1 case when the majority of code pairs have 100% mortality rate. This paper assumed that there was additional one survivor. Then, we calculated the TMR value, and it seemed to decrease death cases. In fact, this modified approach is appropriate and more in conformity with clinical practice.

This study uses TMR, NBR, and BR to create three separate probit regression models respectively for the specific ICD-10-CM code on different individual patients. Meanwhile, we apply optimal ratio of death probability for NBR and BR to modify the TDP for TMR, in order to acquire optimal value. This is a combination of rigorous statistical regression models and mathematical properties to improve the prediction accuracy. As individual’s contribution to the death depends mainly on the three most severe traumas such as ISS, NISS, and TISS agents that have been confirmed, on a specific ICD-10-CM code using different individual patients, the three largest TDP weighted median as its final value (i.e., WMDP) (see Additional file 2).

This study, in IMP-ICDX, when only the death probability value of the most severe injury was used, the coefficient of the worst injury was about four times the coefficient of minor injuries (results not presented). The absolute value of IMP-ICDX and SWI only differs by 0.007, as well as overlapping confidence intervals. What is more, they are still statistically significant (*P* < 0.01), indicating that IMP-ICDX is better than SWI at predicting traumatic death (Table 2). In a sense, SWI model to predict the death is also better [17]. Trauma surgeons usually describe a patient’s clinical condition using the patient’s one or two worst injuries. The TMPM-ICD9 holds that a patient’s five worst injuries determine the possibility of mortality to a great extent [9], because in this dataset, only five coefficients of the most severe injuries in each patient were statistically significant (Table 4). Thus, IMP-ICDX is defined as the sum of the five worst WMDP values. The results greatly improve the accuracy of the predicted death, whether it is calibration, discrimination, or AIC statistics, far better than ISS (Table 2).

We found that the NBR and whether or not the use of mechanical ventilation in injured patients have intrinsic ability and useful parameters in predicting death due to trauma. They are better than patient’s age or gender discrimination. As the existing evaluation methods (e.g., ICISS and TMPM-ICD9) were not involved, we added NBR and ventilator to improve IMP-ICDX trauma result prediction.

In general, additional information (such as respiratory rate, systolic blood pressure, and GCS) to anatomical injury score can always improve the predicted outcomes [4, 9, 18]. The fundamental IMP-ICDX is extremely attractive because only anatomical trauma information is available. IMP-ICDX can also serve as a rich foundation in adding more sophisticated forecasting information to further enhance the accuracy of predicted results. The addition of the ventilator can enhance the AUC of the IMP-ICDX from 0.919 to 0.952 (no analysis). The IMP-ICDX had better discrimination and calibration than the ISS and the SWI models when we added age, gender, and injury mechanism (Table 3).

The goal of this research is to help people predict trauma death probability accurately according to the hospital diagnosis (ICD-10-CM coding), allocate medical resources rationally and effectively, guide clinical diagnosis and treatment, and ultimately improve the efficiency. This unique computing method can be applied to big data processing in other fields, which may lead to a revolutionary era of big data processing.

### Limitations

The main limitation of this article is to inherit defects of the NTDB data. Although the data is bigger, it is not a population-based dataset. In addition, ICD-10-CM coding may have differences because the data is derived from different trauma centers. At the same time, the ICD-10-CM code itself lacks the severity extent of the injury, which is different from the AIS code, and the prediction of the severity of traumatic death is not accurate; it is difficult to determine the injury severity of solid organs in particular, such as the liver, spleen, and kidney. ICD-10-CM codes have 8000 more variables and more than AIS codes, but they are still unable to make up for their defects. As there are too many encoding classifications, the number of single injury code of 60% data is 1988 and 689 codes are lost. If total data is used to calculate WMDP value or to increase the amount of data, the final AUC will be higher. ICD-10-CM-code-based IMP-ICDX outperforms ISS in predicting the death possibility. In this paper, the TMR value is used as a reference only; each TMR is required to be converted to WMDP by combining with the regression models and mathematical characteristics and then evaluating the probability of death of individual patients with different ICD-10-CM codes. Though the process of this calculation method is somewhat complicated, it can improve the ability to predict trauma death. A concurrent cohort study will likely have the same results, and those interested can test our results further.

## Conclusions

In summary, IMP-ICDX is statistically significant compared to ISS, and its predictions of death, discrimination, and calibration are better than those of ISS. Therefore, in our opinion, IMP-ICDX could be a new feasible assessment method for trauma research.

## Supplementary information

**Additional file 1.** The method of calculating TMR. (DOC 29 kb)

**Additional file 2.** Calculating WMDP values. (DOC 48 kb)

**Additional file 3.** Estimating IMP-ICDX. (DOC 22 kb)

**Additional file 4.** WMDP values of different ICD-10-CM injury codes. (XLS 1135 kb)

## Data Availability

The data that support the findings of this study are available from NTDB databases of American College of Surgeons.

## Abbreviations

AIC: Akaike information criterion
AIS: Abbreviated Injury Scale
AUC: Area under the receiver operating characteristic curve
BR: Body region
CI: Confidence interval
GCS: Glasgow Coma Score
H-L: Hosmer-Lemeshow
ICD-10-CM E-codes: International Classification of Diseases Tenth Revision Clinical Modification External cause of injury codes
IMP: Injury mortality prediction
IMP-ICDX: Injury mortality prediction for ICD-10-CM
IQR: Interquartile range
ISS: Injury severity score
ln: Natural logarithm
MARC: Model-averaged regression coefficient
MMR: Multiple injuries mortality rate
NBR: Number of body region
NISS: New injury severity score
NTDB: National Trauma Data Bank
SMR: Single injury mortality rate
SRR: Survival risk ratio
SWI: Single worst injury
TDP: Trauma death probability
TISS: Tangent injury severity score
TMPM: Trauma mortality prediction model
TMPM-ICD9: Trauma mortality prediction model for ICD-9-CM
TMR: Trauma mortality rate
WMDP: Weighted median death probability
