# Supplementary material for: IMP-ICDX: an injury mortality prediction based on ICD-10-CM codes
Source: World J Emerg Surg. 2019 Oct 11;14:46. doi: 10.1186/s13017-019-0265-y (PMC6787998; doi:10.1186/s13017-019-0265-y)
Supplement: Supplementary file 1 — Additional file 1. The method of calculating TMR. (DOC 29 kb) [file 13017_2019_265_MOESM1_ESM.doc]

**Additional file 1**

**The method of calculating TMR**

According to the functional relationship between each age group and their corresponding crude death rate for the population in the United States in 2015, [[16](#OLE_LINK16)] their trend line function: $y=0.01252\times e^{0.0715\times age}$ (*e* = 2.71828…), the correlation coefficient (R^2^) is 0.9817. The corresponding possible mortality rate (PMR, *y*) can be calculated according to different ages (from 1 to 89 years old). PMR can be used as a value that the actual mortality rate for different ICD-10-CM codes equals to zero. By way of example, a 49-year-old patient, PMR = 0.01252 × *e* ^0.0715 × age^ = 0.4160591 (%). The study introduces the gold split ratio of 0.618 as a parameter. Because not every injury contributes to death.

This text used 60% of the data to assess trauma mortality rate (TMR). First, calculate the single injury mortality rate (SMR). In a single injury, the specific ICD-10-CM injury code death number (D1) was divided by the total number of cases (T1), and then multiplied by 100, that is SMR = D1 / T1 × 100. If D1 = 0, this study sets the mortality rate as the median of PMR (PMR_M) divided by the total number of cases (T1), SMR = PMR_M / T1. If D1 = T1, we assume that a survivor case is added, SMR = D1 / (T1+1) × 100.

Second, calculate multiple injury mortality rate (MMR) (with an average of 4.891 lesions per multiple injured patient in this study). In patients with multiple injured, the number of specific ICD-10-CM injury code death (D2) was divided by the total number of injured incidents (T2) multiplied by 100 and then divided by 4.891, MMR = D2 / (T2 × 4.891 × 0.618) × 100. If D2 = 0, this study sets the mortality rate as the median of PMR (PMR_M) divided by 4.891 and the total number of cases (T2), MMR = PMR_M / (T2 × 4.891 × 0.618). If D2 = T2, we assume that a survivor case is added, MMR = D2 / ((T2+1) × 4.891× 0.618) × 100.

Third, merge single and multiple injury mortality rates of specific trauma ICD-10-CM code, TMR = (T1 × SMR + T2 × MMR) / (T2 + T1). In order to ensure that each TMR is different, the same TMR values in different ICD-10-CM codes, TMR is reduce the 0.0000001 in turn according to the size of the sum of T2 and T1 and different ICD-10-CM codes in this study. There is a total of 8,534 TMR values for different trauma ICD-10-CM codes listed in Additional file [4](../4.%20Additional%20file%204.xls).xls.

TMR indicates the trauma mortality rate of specific ICD-10-CM code. SMR indicates single mortality rate of specific trauma ICD-10-CM code. MMR shows multiple injury incidents mortality rate of specific trauma ICD-10-CM code. D1 and D2 represent the number of single deaths and multiple deaths for specific trauma ICD-10-CM code respectively. T1 and T2 indicate the total number of single trauma cases and multiple traumatic incidents for specific trauma ICD-10-CM code. PMR is a possible mortality rate of single and multiple for specific trauma ICD-10-CM code. PMR_M is a median of PMR for specific trauma ICD-10-CM code.
