# Supplementary material for: IMP-ICDX: an injury mortality prediction based on ICD-10-CM codes
Source: World J Emerg Surg. 2019 Oct 11;14:46. doi: 10.1186/s13017-019-0265-y (PMC6787998; doi:10.1186/s13017-019-0265-y)
Supplement: Supplementary file 2 — Additional file 2. Calculating WMDP values. (DOC 48 kb) [file 13017_2019_265_MOESM2_ESM.doc]

**Additional file 2**

**Calculating WMDP values**

This text sets 7 body regions (BR) (1, Head and neck; 2, Face; 3, Chest; 4, Abdomen; 5, Extremities; 6, Skin and 7, Other). Any injury body regions that cannot be classified as from 1 to 6 are all set to 7, such as ICD-10-CM code, T141, “Open wound of unspecified body region” and ICD-10-CM code, T79.4XXA, “Traumatic shock, initial encounter”, etc.

TMR value replaces each injury ICD-10-CM code (Additional file [4](../4.%20Additional%20file%204.xls).xls). This study mainly adopts 8,534 TMR values of possible injury ICD-10-CM codes, NBR and BR as fundamental predictors to created three separate probit regression models, respectively. Comprehensively 6 additional variables were employed in three models to decrease the variance: age (as age^3^ and age^3^ × ln(age), suggested by fractional polynomial analysis [[13](#OLE_LINK13)]), gender, ventilator, injury mechanism, GCS motor component and hospital fixed effects. The mathematical expressions were as follows:

$$\begin{matrix} P(death)1=\Phi\left(\alpha_{0}+\alpha_{1}\sum_{r=1}^{8534} TMR_{r}+\alpha_{2}\sum_{i=3}^{15} (16-GCS_{i})+\alpha_{3}(age^{3})\ln(age)\right. \\ \left.+\alpha_{4}age^{3}+\alpha_{5}ventilator+\alpha_{6}gender+\alpha_{7}\sum_{j=1}^{6} mechanism_{j}+\alpha_{8}\sum_{k=1}^{738} H_{k}\right) \end{matrix}$$

$$\begin{matrix} P(death)2=\Phi\left(\beta_{0}+\beta_{1}\sum_{s=1}^{50} NBR_{s}+\beta_{2}\sum_{i=3}^{15} (16-GCS_{i})+\beta_{3}(age^{3})\ln(age)\right. \\ \left.+\beta_{4}age^{3}+\beta_{5}ventilator+\beta_{6}gender+\beta_{7}\sum_{j=1}^{6} mechanism_{j}+\beta_{8}\sum_{k=1}^{738} H_{k}\right) \end{matrix}$$

$$\begin{matrix} P(death)3=\Phi\left(\gamma_{0}+\gamma_{1}\sum_{t=1}^{7} BR_{t}+\gamma_{2}\sum_{i=3}^{15} (16-GCS_{i})+\gamma_{3}(age^{3})\ln(age)\right. \\ +\gamma_{4}\left.age^{3}+\gamma_{5}ventilator+\gamma_{6}gender+\gamma_{7}\sum_{j=1}^{6} mechanism_{j}+\gamma_{8}\sum_{k=1}^{738} H_{k}\right) \end{matrix}$$

where *P*(death)1, *P*(death)2, and *P*(death)3 are the mortality predicted by the *P*_TMR_, *P*_NBR_, and *P*_BR_, respectively and Φ is the standard normal cumulative distribution function.

TMR*_r_*, *r* = 1, ..., 8,534 is a binary indicator variable for each of the 8,534 modified ICD-10-CM codes.

NBR*_s_*, *s* = 1, ..., 50 is a binary indicator variable for each of the 50 NBR values.

BR*_t_*, *t* = 1, ..., 7 is a binary indicator variable for each of the 7 BRs.

GCS*_i_*, *i* = 3, ..., 15 is a binary indicator variable for each of the 13 GCS values.

mechanism*_j_*, *j* = 1, ..., 6 represents a binary indicator variable for the mechanism of injury.

H*_k_*, *k* = 1, ..., 738 is a binary indicator variable for each of the 738 hospitals.

Among 60% of data set that are used to develop *P*_TMR_, *P*_NBR_, and *P*_BR_, there are 40 ICD-10-CM codes lacking GCS values. The median value of the original GCS was taken as their values (GCS of the dead was 5, and that of the survivors was 15) to make up for missing GCS values, according to whether the patient died at the time of discharge. In this study, as for the patients who needed mechanical ventilation, Ventilator Days (an independent variable) were set to 1, 0 otherwise.

We then combine four variables: *P*_TMR_, *P*_NBR_, *P*_BR_, and TMR to estimate each traumatic death probability (TDP) value: where “ln” indicates natural logarithm. In order to ensure that each variable is not negative, the corresponding constants (3.91, 4.14, and 3.93) are added to *P*_TMR_, *P*_NBR_, and *P*_BR_, respectively.

This paper estimates the weighted median of the three worst (greatest) TDP values for each injury ICD-10-CM code, namely, which is called Weighted Median Death Probability (WMDP). The formula is as follow:

$WMDP_{v}=TDP_{\frac{N_{v}+1}{2}}$, when N_v_ is an odd number.

$WMDP_{v}=\frac{TDP_{N_{v}/2}+TDP_{N_{v}/2+1}}{2}$, when N_v_ is an even number.

where N_v_ is the number of the three worst (greatest) TDP values or whatever are lesser than the third worst TDP value in specific ICD-10-CM code.

In this study, 60% data was used to develop 8,534 WMDPs (Additional file [4](../4.%20Additional%20file%204.xls).xls), the median value of 2,748 TDPs (32.2%, only 0.3% of the BR involved) in ICD-10-CM codes was equal to the mean, and each code had only 1 or 2 injuries. The median of 3,740 TDPs (43.8% and the involved BR accounting for 72.3%) was lesser than the mean and the rest were greater than the mean. From above, most data in this study was non-normal distribution, so that the median of TDP was taken as the final value (i.e. WMDP).
