# Supplementary material for: IMP-ICDX: an injury mortality prediction based on ICD-10-CM codes
Source: World J Emerg Surg. 2019 Oct 11;14:46. doi: 10.1186/s13017-019-0265-y (PMC6787998; doi:10.1186/s13017-019-0265-y)
Supplement: Supplementary file 3 — Additional file 3. Estimating IMP-ICDX. (DOC 22 kb) [file 13017_2019_265_MOESM3_ESM.doc]

**Additional file 3**

**Estimating IMP-ICDX**

It is necessary to replace IMP-ICDX codes with their respective WMDP values (see Additional file [4](../4.%20Additional%20file%204.xls).xls). WMDP values are sorted by the severity among patients who sustained multiple injuries. We apply 20% of the data (not used as a development of WMDP) to estimate the coefficient values of IMP-ICDX with the logistis regression method. In order to assess the IMP-ICDX probability of death for an individual patient, the following formula is deduced: $\begin{matrix} P\left(death\right)=\Lambda\left(C_{0}+\sum_{i=1}^{5} C_{i}\times I_{i}+C_{6}\times I_{1}\times I_{2}\right.+C_{7}\times S \\ \left.+C_{8}\times ln\left(NBR\right)+C_{9}\times NBR^{0.382\mathop{}\limits}\right) \end{matrix}$

where *P*(death) is the mortality predicted by the IMP-ICDX and Λ is the cumulative distribution function for the logistic distribution, and *I*_1_, ..., *I*_5_ are the WMDP values for the 5 worst injuries, ordered with the highest WMDP value (worst injury) first, the second highest WMDP value second, up to the fifth worst injury. *I*_1_×*I*_2_ represents the interaction of the WMDP values for the worst two injuries. S is equal to 1 if the two worst injuries are in the same body region, 0 otherwise. “ln” indicates natural logarithm. NBR is the number of body regions in each injured patient. *C*_0_, ..., *C*_9_ are coefficients in Table [4](../2.%20Table%204.doc).

The authors will provide an IMP-ICDX calculator available to the Microsoft Ecxel platform (see Additional file [4](../4.%20Additional%20file%204.xls).xls). In fact, the IMP-ICDX can also be calculated by any computer language.
